# Multifocal Gastric Granular Cell Tumor: A Case Report

**DOI:** 10.34172/mejdd.2024.400

**Published:** 2024-10-30

**Authors:** Samira Saeian, Kamran B Lankarani, Mohammad Hossein Anbardar, Seyed Majid Ahmadi

**Affiliations:** ^1^Gastroenterology and Hepatology Research Center, Shiraz University of Medical Sciences, Shiraz, Iran; ^2^Health Policy Research Center, Health Institute, Shiraz University of Medical Sciences, Shiraz, Iran; ^3^Department of Pathology, Shiraz University of Medical Sciences, Shiraz, Iran

**Keywords:** Granular cell tumors, Multifocal, Stomach

## Abstract

Granular cell tumors (GCTs) of the gastrointestinal tract are rare neoplasms often detected incidentally as subepithelial lesions during endoscopic examination. The occurrence of GCTs in the gastric cavity is even rarer. So far, there have been only four reports of multifocal gastric GCTs. Our study presents the fifth case involving a middle-aged lady with incidental multifocal gastric GCT. It is the first such case reported in West Asia.

## Introduction

 Granular cell tumors (GCTs) are rare neoplastic lesions originating from Schwann cells, typically occurring more commonly in individuals aged 40 to 60 years, with a slight female predominance.^1^ They typically manifest as small, solitary, nodular growths in various anatomical locations, including the oral cavity, skin, and subcutaneous tissue. The gastrointestinal (GI) tract is an unusual site for GCTs, and the esophagus is the most common location.^2,3^ There are case reports and case series of GCTs in the esophagus, stomach, duodenum, appendix, colon, rectum, and anus.^2-5^

 GCTs are usually asymptomatic and are found incidentally as submucosal lesions during endoscopic examination performed for other reasons. They can simultaneously affect multiple sites in 7%-25% of cases, but only a few cases of multifocal gastric GCTs have been reported.^3,6,7^

 In this article, we present a case of multifocal gastric GCT incidentally discovered in a patient who presented with nausea and vomiting and was subsequently diagnosed with adrenal insufficiency.

## Case Report

 A 45-year-old woman presented to the emergency department with worsening abdominal pain, nausea, and vomiting in the previous 3 days. She reported experiencing progressive epigastric pain, nausea, and vomiting for 3 weeks, accompanied by fatigue, anorexia, and weight loss. She denied any diarrhea, hematemesis, or melena.

 Physical examination was unremarkable except for darkening of the skin, especially on the face and in skin folds.

 She had a history of convulsions, for which she had been taking valproate sodium for 2 years, hypothyroidism treated with levothyroxine, and secondary amenorrhea. Initial laboratory tests revealed normocytic anemia with a normal iron profile. Serum amylase and lipase were within normal limits. However, an ultrasound of the abdomen showed evidence of gallbladder sludge.

**Figure 1:**
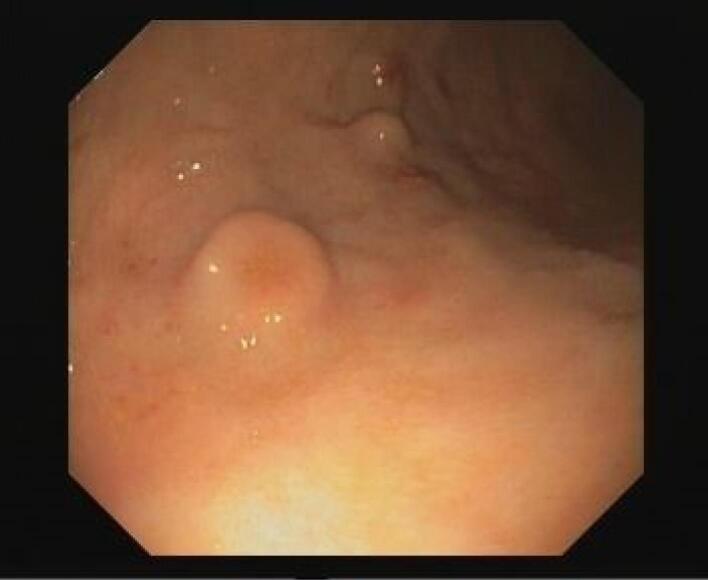
Upper endoscopy showing a mass-like lesion with central depressive mucosal ulceration on the surface

 Further investigation with upper endoscopy demonstrated three mass-like lesions with central depressive mucosal ulceration on the surface. The lesion biopsy at that time was non-diagnostic (Figure 1).

**Figure 2:**
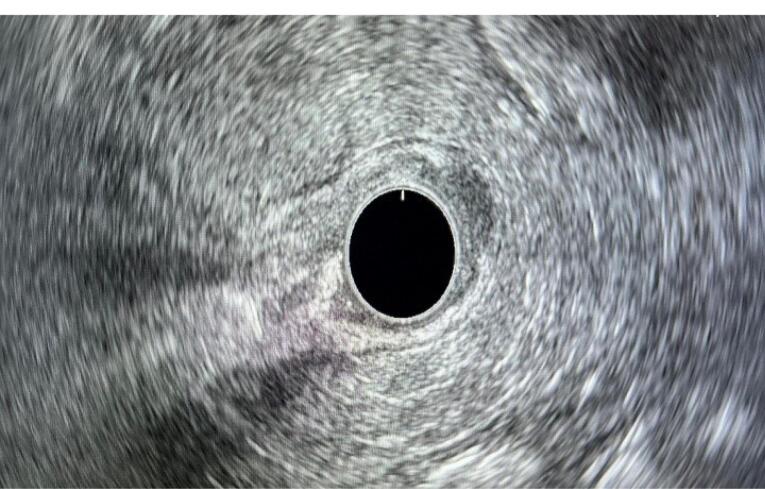
Round hypoechoic lesions within the submucosa were observed on endoscopic ultrasound

 The contrast-enhanced computed tomography (CT) of the chest and abdomen was unremarkable except for mild wall thickening of the antrum. Round hypoechoic lesions within the submucosa were observed on the endoscopic ultrasound (EUS) of the gastric lesions (Figure 2). The largest lesion measured 13 mm in the largest diameter at the proximal lesser curvature. The other two lesions were 4 mm distal to the previous lesion and 10 mm in the antrum with similar characteristics.

**Figure 3:**
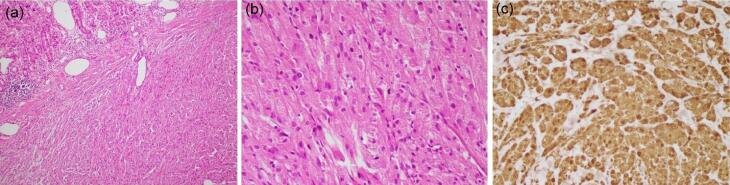
**(a)** Low-power view of gastric submucosal mass (Hematoxylin and eosin, × 100). **(b)** Microscopic section showing polygonal and spindled cells with abundant and coarsely granular cytoplasm (Hematoxylin and eosin, × 400. **(c)** Immunoreactivity for S100 ( × 400)

 The lesions were removed using the endoscopic submucosal dissection hybrid technique. Histopathological examination revealed a well-defined rubbery mass beneath the gastric mucosa. Microscopic evaluation showed polygonal to slightly spindled cells with relatively distinct cell borders and abundant eosinophilic and granular cytoplasm (Figures 3a and 3b). Immunohistochemistry analysis demonstrated strong cytoplasmic and nuclear positivity for S100 (Figure 3c) and cytoplasmic positivity for CD68 and CD56, consistent with diagnosing GCTs.

 Subsequent evaluation based on physical examination and laboratory data led to a presumed diagnosis of adrenal insufficiency, which was later confirmed with a low serum cortisol level and a high ACTH level. There was no response to ACTH stimulation. Concurrently, elevated TSH levels with positive anti-TPO antibodies were indicative of primary adrenal insufficiency in the presence of autoimmune polyglandular syndrome type 2. Treatment with hydrocortisone resulted in gradual improvement of the symptoms.

## Discussion

 In 1926, Abrikosoff ﬁrst described GCTs as ‘granular cell myoblastoma’ in an oral lesion because of their suspected muscle origin.^7^ However, subsequent molecular studies have identified Schwann cells as their origin.^1^

 The GI tract is an uncommon site for GCTs, constituting only 8% of all cases. The esophagus is the most common site, followed by the colon, perianal region, stomach, small intestine, and appendix.^5^

 More than 70 cases of gastric GCTs have been reported in the literature, mainly in the fourth to sixth decade of life, with no sex predisposition.^8^ However, female dominance has been reported in some series.^1^ Most cases of gastric GCTs are reported among East Asian and African-American ethnicities.

 Endoscopically, GCTs are sessile, small (usually less than 20 mm), and submucosal with a whitish appearance, sometimes exhibiting ulceration similar to molars on the gingiva.^4^

 However, distinguishing GCTs from other submucosal tumors, such as GI stromal tumors or carcinoids, based on endoscopic appearance alone can be challenging.

 EUS is the best tool to characterize subepithelial lesion (SEL) features (i.e., size, location, originating layer, echogenicity, and shape), although it cannot alone distinguish among all SEL types.^9^

 EUS with tissue acquisition will improve accuracy in solid non-lipomatous SEL diagnosis. However, the unroofing technique is necessary when EUS-guided fine-needle aspiration or fine-needle biopsy is non-diagnostic.^10^

 In the evaluation of GCTs, EUS reveals hypoechoic, mildly inhomogeneous, well-defined lesions arising from the second or third mucosal layer (deep mucosa or submucosa).^11^

 Clinically, most GCTs are detected incidentally and are benign, with a malignancy rate estimated to be less than 2%.^1^

 Microscopically, GCTs are characterized by sheets or nests of large, pale, ovoid cells with a poorly deﬁned cellular membrane and granular, eosinophilic cytoplasm. Cytoplasmic granules contain large amounts of hydrolytic enzymes with scattered giant lysosomal granules.

 Immunohistochemically, GCTs show evidence of schwannian diﬀerentiation, with positivity for S100. Most GCTs also express SOX10, and some can be positive for CD68, calretinin, and inhibin.^12,13^

 S100 is a valuable tool for differentiating GCTs from other submucosal lesions, which are usually S100 negative.

 The six histological criteria to evaluate the malignant potential of GCTs, as described by Fanburg-Smith, are necrosis, spindling, vesicular nuclei with large nucleoli, increased mitotic activity, high nuclear-to-cytoplasmic ratio, and pleomorphism.^13^

 Surveillance of asymptomatic GI tract leiomyomas, lipomas, heterotopic pancreas, GCTs, schwannomas, and glomus tumors is not recommended if the diagnosis is precise.^9^

 Most GCTs are solitary lesions; however, concomitant lesions in other parts of the GI tract, especially the esophagus, are observed in 50% of cases.^1,6,11,12^ Our case showed no concomitant lesions in the esophagus and colon.

 To our best knowledge, only four cases of multiple gastric lesions have been reported in the literature among more than 70 cases of gastric GCTs,^14,15^ and our patient represents the fifth case.

 We did not find any association between GCTs and autoimmune disorders in the literature, and the co-occurrence of polyglandular failure in this case could be incidental.

 Asymptomatic small lesions of less than one centimeter can be observed. Larger lesions need resection, which can be performed endoscopically.^3^ Our case was treated with endoscopic submucosal dissection.

## Conclusion

 In conclusion, we present a case of multifocal gastric GCT in a middle-aged woman with concomitant autoimmune polyglandular failure who was treated with endoscopic resection. Gastric GCTs should be considered in the differential diagnosis of subepithelial lesions in the stomach, even when they are multiple and without concomitant esophageal lesions.
